# Trends in sugar content of non-alcoholic beverages in Australia between 2015 and 2019 during the operation of a voluntary industry pledge to reduce sugar content

**DOI:** 10.1017/S1368980022002300

**Published:** 2022-10-24

**Authors:** Ana-Catarina Pinho-Gomes, Elizabeth Dunford, Alexandra Jones

**Affiliations:** 1 School of Life Course & Population Sciences, Faculty of Life Sciences & Medicine of Population, King’s College London, London, UK; 2 The George Institute for Global Health, Imperial College London, 84 Wood Lane, London W12 0BZ, UK; 3 The George Institute for Global Health, University of New South Wales, Sydney, NSW, Australia; 4 Department of Nutrition, Gillings Global School of Public Health, The University of North Carolina, Chapel Hill, USA

**Keywords:** Sugar-sweetened beverages, Diet, Food policy, Health policy

## Abstract

**Objectives:**

To investigate changes in mean sugar content of non-alcoholic beverages (overall and sugar-sweetened beverages (SSB)) available for purchase in Australia and to compare signatories *v*. non-signatories of the Australian Beverages Council voluntary pledge from 2018

**Design:**

Retrospective observational study.

**Setting:**

Australia.

**Participants:**

About 1500 non-alcoholic beverages per year included in the FoodSwitch Monitoring Datasets for 2015–2019.

**Results:**

Overall, mean sugar content fell by 1·3 g/100 ml (17·1 %) from 7·5 g/100 ml in 2015 to 6·2 g/100 ml in 2019. SSB have accounted for about 56 % of all beverages available for purchase since 2015. Between 2015 and 2019, the sugar content of SSB dropped by about 10 % (0·8 g/100 ml). Soft drinks and milk-based drinks were the categories with the largest decrease in sugar content. The greater reduction in sugar observed for beverages overall than SSB suggests at least some of the overall decrease in sugar content is due to the appearance of new products with low or no sugar rather than reformulation. Over the same period, beverages with added non-nutritive sweeteners increased from 41 % to 44 %. The decrease in sugar content for all beverages and SSB was, in general, larger for non-signatories than signatories of the voluntary industry pledge.

**Conclusions:**

Between 2015 and 2019, the small reduction in sugar content of non-alcoholic beverages in Australia resulted from the combined effects of introducing low- or no-sugar products and reformulation of some categories of SSB. Further policy and regulatory measures are required to reap the most benefit that sugar reduction among non-alcoholic beverages can bring to population health.

In Australia, 25 % of children and adolescents and 67 % of adults are living with overweight or obesity^(1)^. This burden is experienced disproportionately in lower socio-economic groups, those living in rural and remote areas, and by First Nations Australians^(1)^. Evidence suggests that this may be partially explained by excessive sugar intake, particularly if consumed in liquid form^(2)^. Indeed, the most recent Australian National Nutrition Survey showed that Australians consumed an average of 60 g of free sugars per d (equivalent to fourteen teaspoons of white sugar) in 2011–2012, which albeit high represented a reduction in dietary energy from free sugars from 12·5 % in 1995 to 10·9 % of energy in 2011–2012^(3)^.

Excessive intake of free sugars has been associated not only with overweight/obesity in children and adults^(4–6)^ but also cardiometabolic diseases^(7,7,8)^ and dental caries^(9)^. Furthermore, randomised trials showed reduced intake of dietary sugars was associated with an about 0·8 kg decrease in body weight, but isoenergetic exchange of dietary sugars with other carbohydrates showed no change in body weight^(10)^. However, trials in children, which involved recommendations to reduce intake of sugar-sweetened foods and beverages, had low participant compliance to dietary advice and showed no overall change in body weight^(10)^.

Sugars are carbohydrates that occur naturally in foods like milk and whole fruits but also appear in a processed form throughout the food supply. Existing Australian Dietary Guidelines recommend avoiding ‘added’ sugars, whereas more recent WHO guidance recommends limiting consumption of ‘free’ sugars, which include both ‘added’ sugars and sugars naturally present in honey, syrups, fruit juices and fruit juice concentrates, recognising the equally harmful impacts of these sugars on the body^(11,12)^. Current intake guidelines from WHO suggest that free sugars should account for <10 % of total energy intake (approx. 53 g/13 teaspoons) to prevent unhealthy weight gain and dental caries^(12)^. Australian adults consume, on average, fourteen teaspoons of free sugars daily, with adolescents and young adults consuming far more^(13)^. More than half (52 %) of free sugars in the Australian diet derive from beverages, particularly soft drinks, sports and energy drinks (19 %), fruit and vegetable juices (13 %) and cordials/syrups (4·9 %)^(13)^.

Given their substantial contribution to excess sugars consumption and otherwise limited nutritional value, governments around the world are taking policy action to reduce intake of sugar-sweetened beverages (SSB). At a global level, WHO recommends effective taxation of SSB among its ‘best-buy’ policies to address non-communicable diseases^(14)^. Despite widespread support from public health and consumer groups^(15)^, there has been limited political appetite for a similar levy on SSB in Australia. Instead, national action has been limited to a voluntary ‘Health Food Partnership’ (HFP) established by the Australian Federal Government in 2015, which included targets for sugar reduction to be achieved by 2025^(16)^.

Outside of government initiatives, the Australian Beverages Council (ABC), the peak industry group representing makers of non-alcoholic beverages, has announced their own voluntary ‘Sugar Reduction Pledge’^(17)^. Although announced in 2018, it purported to apply retroactively from 2015. Signatories of the ABC pledge committed to reducing sugar content across their non-alcoholic beverage portfolios by 20 % between 2015 and 2025, measured through sales-weighted average reductions in total grams of sugar per 100 ml across their portfolios. To date, four companies have become signatories: Asahi Lifestyle Beverages, Coca-Cola South Pacific, Coca-Cola Amatil and PepsiCo, which together account for over 80 % of the volume of sales of non-alcoholic beverages in Australia. Reductions can be achieved by a variety of actions including reformulating existing products, introduction of low- or no-sugar varieties, increasing sales of low- or no-sugar varieties and introducing smaller pack sizes. The ABC appointed consulting firm KPMG as an independent aggregator of industry self-reported data on progress. However, a peer-reviewed evaluation of progress towards the pledge is lacking.

To inform ongoing discussion about the need for stronger policy and/or regulatory action, the primary aim of this study is to investigate changes in the sugar content of non-alcoholic beverages available for purchase in Australia between 2015 and 2019. As secondary aims, we also analyse changes in the presence of sugar substitutes, also called non-nutritive sweeteners (NNS), in these products over the same period, and examine results separately for the four signatory companies to the ABC pledge.

## Methods

### Data source

The George Institute’s FoodSwitch programme captures images of packaged foods and beverages using a smartphone application^(18,19)^. Using this process, the FoodSwitch Monitoring Datasets are generated annually based on systematic data collection from four large Australian supermarket stores (Aldi, Coles, Independent Grocers of Australia and Woolworths) in the Sydney metropolitan area. Data collection takes place over a 4-month period each year. Trained data collectors take images of products in-store, capturing information including product barcode, product name, package size, ingredients list, manufacturer and brand names, and nutrition information on the front and back of pack. The database represents >90 % of the Australian packaged food and beverage market^(18)^. The 2015, 2016, 2017, 2018 and 2019 FoodSwitch Monitoring Datasets were used in this study.

### Beverages included in this analysis

Using the categorisation system developed by the Global Food Monitoring Group, foods and beverages in the FoodSwitch programme are classified into a hierarchical tree to allow for comparison of nutritionally similar foods^(19)^. Products are categorised into major, minor and further subcategories. Our analysis included packaged non-alcoholic beverage products from the following categories: cordials/syrups, electrolyte/sports drinks, energy drinks, fruit and vegetable juices, soft drinks (including iced teas), waters, breakfast beverages, milks, and milk-based protein drinks. We included coffee products that were ready-to-drink (e.g. iced coffee milks) but excluded plain coffee and coffee products that required further preparation. We also excluded other beverage powders and mixes that can be made up in a variety of preparations (e.g. hot chocolates), and all yogurts and probiotic drinks. A detailed description of the included categories and their definitions is provided in Supplemental Table S1.

For each beverage, we extracted the following information: product name, manufacturer’s name, total sugar content in grams per 100 ml, full ingredients list and category. Where a beverage appeared in more than one package size (i.e. a 375 ml can and 600 ml bottle of the same drink), each package size was counted once (i.e. a 375 ml can and 600 ml bottle were counted as two products).

### Definition of sugar-sweetened beverages

To generate deeper insight into the extent that reformulation contributed to potential changes in sugar content (as opposed to introducing low- or no-sugar alternatives), we conducted additional analysis of changes in the sugar content of SSB specifically. For this purpose, SSB were defined as drinks containing added sugar ingredients. The full list of search terms used for added sugars is provided in Supplemental Table S2. For the purposes of this analysis, fruit juice concentrate or equivalents were not considered as added sugars when present in beverages, in keeping with the current law in Australia on making a ‘no added sugar’ claim^(20)^. However, sensitivity analyses performed considering fruit juice concentrate and equivalent as added sugars had no material impact of the findings of this study and are available upon request.

### Presence of non-nutritive sweeteners

To complement our analysis of changes in sugar content, we also analysed changes in the presence of NNS in beverages. As NNS are added in very small quantities and this amount is not required to be disclosed on the nutrition information panel, this was defined as a binary variable as presence or absence of NNS. Products were classified as containing NNS based on keyword searches within the ingredient list for each product. A detailed list of NNS terms was compiled based on reviewing food labelling and additive regulations relating to sweeteners in Australia. Search terms for NNS are provided in Supplemental Table S3. Search terms are those that are listed as intense sweeteners under Food Standards Australia New Zealand nomenclature.

### Data analysis

First, to demonstrate trends in the availability of beverages in the Australian food supply, we calculated the number of products in each beverage category between 2015 and 2019. Second, to investigate changes in total sugar content, we calculated absolute and percentage change in total sugar content per 100 ml in all beverages between 2015 and 2019. In addition, we calculated the changes in sugar content between 2015 and 2018 to understand how much change had occurred prior to the announcement of the ABC pledge. Third, to estimate to whether changes in sugar content were explained by reformulation of existing beverages or addition of sugar-free beverages, we calculated absolute and percentage change in total sugar content per 100 ml for SSB. Fourth, to investigate whether sugar reductions were compensated by increases in NNS, we calculated the proportion of beverages containing at least one NNS. All analyses were stratified by categories of beverages as appropriate.

We carried out subgroup analysis for signatories of the ABC voluntary pledge, compared with those manufacturers who did not sign the pledge. For this subgroup analysis, only categories of beverages that were produced by signatories of the pledge were included. All analyses were carried out using R version 4.0.3.

## Results

A total of 1499, 1503, 1502, 1655 and 1605 beverage products were included for 2015, 2016, 2017, 2018 and 2019, respectively (Supplemental Table S4).

### All beverages

**Fig. 1:**
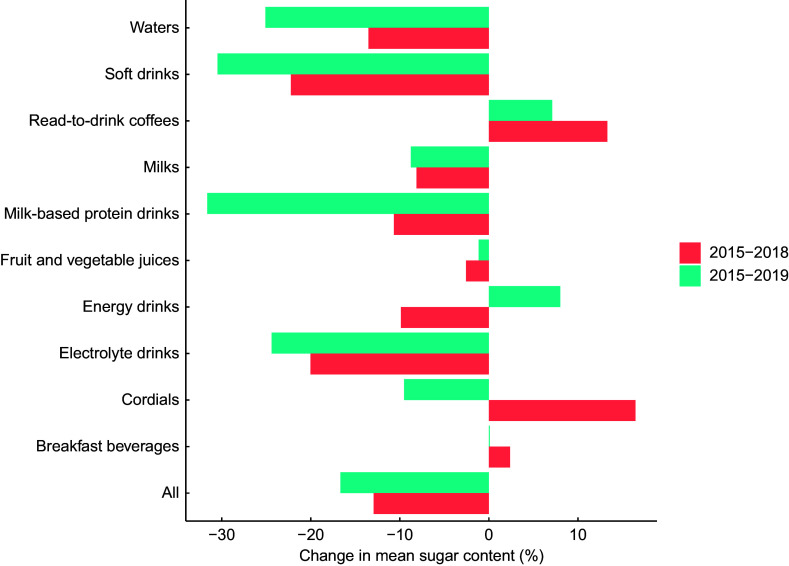
Percentage change in mean sugar content (total sugar in g/100 ml) for all beverages available for purchase in Australia in 2015–2019 and 2015–2018

**Fig. 2:**
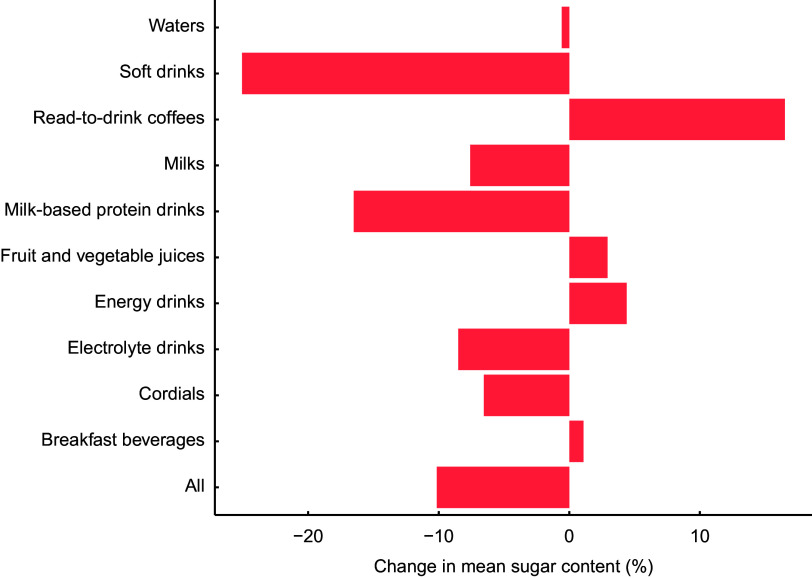
Percentage change in mean sugar content (total sugar in g/100 ml) for sugar-sweetened beverages available for purchase in Australia in 2015–2019

Overall, total sugar content decreased by 1·3 g/100 ml (equivalent to 17·1 %) from 7·5 g/100 ml in 2015 to 6·2 g/100 ml in 2019 (Table 1). However, there was substantial variation between categories, from no change for breakfast beverages to a 2·2 g/100 ml reduction in soft drinks (Fig. 1). For soft drinks and milk-based protein drinks, an almost 30 % reduction was achieved by 2019 (Fig. 2). For ready-to-drink coffees, there was an increase in sugar content from 6·5 to 7·0 g/100 ml.

**Table 1:** Mean sugar content of beverages, stratified by category, available for purchase in Australia between 2015 and 2019

Beverage categories	2015		2016		2017		2018		2019	
	Mean	SD	Mean	SD	Mean	SD	Mean	SD	Mean	SD
Breakfast beverages	7·3	0·9	7·2	1·7	7·2	1·0	7·5	1·3	7·3	1·4
Coffee	6·5	7·5	5·0	2·0	6·6	8·7	7·4	12·0	7·0	11·6
Cordials/syrups	5·7	6·7	6·5	8·8	5·8	3·4	6·6	7·3	5·1	3·7
Sugar-free	0·4	0·4	0·4	0·4	0·5	0·3	0·3	0·2	0·3	0·2
Sugar-sweetened	6·9	6·9	7·9	9·1	6·7	2·8	7·5	7·3	6·3	3·2
Electrolyte drinks	6·2	2·2	5·8	2·5	5·1	2·3	4·9	2·8	4·7	3·0
Sugar-free	0·0	0·0	0·0	0·0	0·0	0·0	0·0	0·0	0·0	0·0
Sugar-sweetened	6·4	1·8	6·4	1·7	6·0	0·6	6·1	1·7	5·9	2·1
Energy drinks	7·4	5·7	7·4	5·4	7·5	5·6	6·6	5·5	8·0	5·6
Sugar-free	0·0	0·0	0·0	0·0	0·0	0·0	0·0	0·0	0·0	0·0
Sugar-sweetened	11·3	2·1	11·0	2·1	11·0	2·9	10·5	2·7	10·6	3·7
Fruit and vegetable juices	9·0	2·7	8·9	2·8	8·9	2·7	8·8	2·8	8·9	2·8
Fruit juice	9·1	2·7	9·0	2·7	9·1	2·7	8·9	2·7	9·0	2·7
Vegetable juice	7·1	2·4	6·8	2·0	6·4	2·2	6·4	2·6	6·9	3·1
Milk	5·8	3·2	5·5	3·0	5·4	3·0	5·3	3·0	5·3	2·9
Coconut milk	1·2	1·7	1·5	1·6	2·7	2·5	2·7	2·1	2·6	1·7
Dairy milk	7·0	2·5	6·8	2·5	6·7	2·5	6·8	2·4	6·6	2·4
Lactose-free milk	5·8	2·0	5·6	1·7	5·5	1·7	6·0	1·8	5·6	1·7
Soya milk	3·8	3·9	3·0	2·1	2·7	2·2	2·9	2·3	2·6	2·0
Other milk	2·7	2·4	2·5	2·0	2·4	1·9	2·7	2·2	2·6	2·0
Milk-based protein drinks	4·5	2·9	4·7	2·7	4·9	2·8	4·0	3·9	3·1	2·1
Soft drinks	7·2	4·7	7·2	4·3	6·8	4·3	5·6	4·7	5·0	4·7
Sugar-free	0·6	1·4	0·6	1·6	0·5	1·7	0·2	0·5	0·2	1·2
Sugar-sweetened	8·8	3·7	8·7	3·2	7·9	3·6	6·7	4·4	6·2	4·5
Waters	3·3	2·8	3·7	6·2	3·4	3·0	2·8	3·0	2·4	2·8
Aloe vera drinks	7·8	3·5	8·7	2·9	8·2	3·9	7·3	3·8	7·0	3·4
Coconut water	4·1	1·1	6·0	9·3	4·5	1·3	4·7	1·4	4·2	1·5
Flavoured waters	3·4	2·1	2·7	2·2	2·7	2·6	1·8	2·4	2·4	2·5
Sparkling water, plain	0·4	1·9	0·4	1·7	0·7	2·3	0·6	2·1	0·2	1·2
Still water, plain	0·0	0·0	0·0	0·0	0·0	0·0	0·0	0·0	0·0	0·0
All	7·5	4·3	7·4	4·5	7·2	4·8	6·6	4·1	6·2	4·8

Values represent mean of total sugar in g/100 ml (SD).

When comparing changes in sugar content from 2015 to 2018 with that from 2015 to 2019, a 20 % reduction in sugar content had already been achieved or surpassed by 2018 for waters, soft drinks, milk-based protein drinks and electrolyte drinks (Fig. 2). Whilst there was a trend towards decreasing total sugar content between 2018 and 2019 for categories such as ready-to-drink coffees and cordials/syrups, for other categories there was minimal progress (e.g. fruit and vegetable juices and milks) or even an increase in sugar content (e.g. energy drinks) between 2018 and 2019 (Fig. 2).

### Sugar-sweetened beverages

About 56 % of beverages across all categories in 2019 contained added sugars as ingredients, and this percentage was relatively stable from 2015 onwards (Table 2). In 2019, the categories with the highest proportion of beverages containing added sugar were breakfast beverages (94 %), soft drinks (89 %), ready-to-drink coffees (85 %) and aloe vera drinks (85 %). Fruit and vegetable juices contained the lowest proportion of products containing added sugar (25 %). Categories with the largest reduction in the proportion of products between 2015 and 2019 containing added sugar ingredients were flavoured waters (from 73 % to 56 %), electrolyte drinks (from 92 % to 80 %) and ready-to-drink coffees (from 95 % to 85 %). No categories showed an increase in the proportion of products containing added sugar ingredients between 2015 and 2019.

**Table 2:** Percentage of beverages that were sugar-sweetened, stratified by category, available for purchase in Australia between 2015 and 2019

Beverage categories	2015	2016	2017	2018	2019
Breakfast beverages	100·0	96·2	100·0	100·0	93·5
Ready-to-drink coffees	95·1	92·9	91·8	87·1	85·2
Cordials/syrups	77·8	81·1	83·8	86·6	76·8
Electrolyte drinks	92·3	89·7	80·6	80·9	79·1
Energy drinks	70·0	65·2	66·0	58·7	68·8
Fruit and vegetable juices	27·7	25·8	25·3	25·4	24·9
Milks	51·4	49·6	51·4	50·8	49·8
Milk-based protein drinks	66·7	61·1	62·5	51·4	30·0
Soft drinks	92·4	94·8	95·7	94·7	89·1
Aloe vera drinks	87·5	88·9	80·0	81·8	84·6
Coconut water	28·6	35·0	29·1	28·6	21·4
Flavoured waters	72·7	62·5	48·9	41·5	55·8
Total	56·3	55·4	55·1	57·3	55·8

Overall, there was a 9·8 % reduction in the mean sugar content of SSB from 8·1 g/100 ml in 2015 to 7·3 g/100 ml in 2019 (Table 3). The largest reductions in sugar content were observed for soft drinks (from 9·2 g to 6·9 g/100 ml) and milk-based protein drinks (from 5·6 g to 4·6 g/100 ml). There was an increase in sugar content for ready-to-drink coffees from 6·7 g to 7·8 g/100 ml. Sugar content remained relatively stable for the remaining categories.

**Table 3:** Mean sugar content of sugar-sweetened beverages (total sugar in g/100 ml), stratified by category, available for purchase in Australia between 2015 and 2019

Beverage categories	2015		2016		2017		2018		2019	
	Mean	SD	Mean	SD	Mean	SD	Mean	SD	Mean	SD
Breakfast beverages	7·3	0·9	7·0	1·4	7·2	1·0	7·5	1·3	7·4	1·4
Ready-to-drink coffees	6·7	7·5	5·2	1·7	7·1	8·9	8·1	12·6	7·8	12·2
Cordials/syrups	7·1	6·9	8·0	9·1	6·8	2·7	7·6	7·3	6·6	3·0
Electrolyte drinks	6·4	1·8	6·4	1·6	6·0	0·6	5·7	2·1	5·9	2·0
Energy drinks	10·9	3·0	10·9	2·1	11·4	2·1	11·1	1·7	11·4	2·6
Fruit and vegetable juices	9·4	2·7	9·6	2·7	9·7	2·7	9·6	2·6	9·7	2·6
Milks	7·3	3·6	6·9	3·4	6·9	3·2	6·9	3·1	6·8	3·2
Milk-based protein drinks	5·6	2·9	6·0	2·6	5·7	2·9	4·4	2·5	4·6	2·1
Soft drinks	9·2	3·5	9·0	3·0	8·0	3·6	7·0	4·3	6·9	4·3
Aloe vera drinks	8·8	2·6	9·7	1·1	10·1	0·6	8·8	2·4	8·2	2·1
Coconut water	5·4	0·8	5·5	1·0	5·8	0·9	5·8	0·9	6·0	0·7
Flavoured waters	4·2	1·5	4·0	1·6	4·9	1·4	3·8	2·2	3·9	2·3
Total	8·1	4·3	8·0	4·2	7·8	3·9	7·3	5·1	7·3	4·7

Values represent mean of total sugar in g/100 ml (SD).

### Non-nutritive sweeteners

Between 2015 and 2019, there was a 3 % overall increase in the percentage of beverages with at least one NNS from 41·2 % to 44·3 % (Supplemental Table S5). This increase was particularly marked for waters (from 3·4 % to 13·2 %) and electrolyte drinks (from 11·5 % to 32·6 %). There was a small reduction in the percentage of beverages with at least one NNS for breakfast beverages, ready-to-drink coffee and cordials/syrups.

### Pledge signatories *v*. non-signatories

**Fig. 3:**
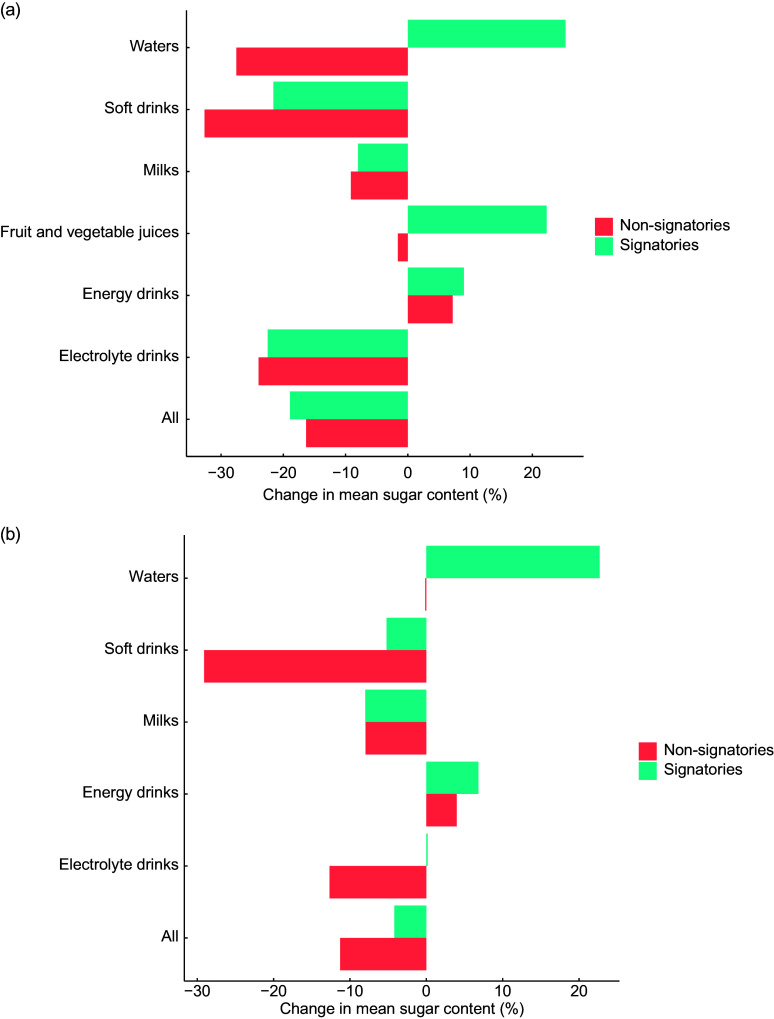
Percentage change in mean sugar content (total sugar in g/100 ml) for pledge signatories *v*. non-signatories between 2015 and 2019 for all beverages (a) and sugar-sweetened beverages (b)

Between 2015 and 2019, the absolute reduction in sugar content for all beverages was larger for non-signatories than signatories of the industry pledge (19 % *v*. 16 %, respectively; Fig. 3(a)). For all beverage categories except energy drinks, there was an absolute reduction in sugar content for non-signatories of the pledge. For pledge signatories, there was an absolute reduction in sugar content for soft drinks, milks and electrolyte drinks, and an increase in sugar content for waters, fruit and vegetable juices and energy drinks. When considering only SSB, the absolute reduction in sugar content was still greater for non-signatories than signatories of the pledge (11 % *v*. 4 %, respectively) (Fig. 3(b)). The largest reductions were observed for electrolyte and soft drinks.

Between 2015 and 2018, when the pledge was signed, both signatories and non-signatories had already reduced total sugar content across all categories of beverages, albeit to variable extents (Supplemental Fig. S2A). Overall, the decrease in sugar content was more marked for non-signatories than signatories of the pledge (16 % *v*. 13 %, respectively). The reduction in sugar content was also greater among non-signatories than signatories of the pledge for SSB (11 % *v*. 2 %, respectively; Supplemental Fig. S2B).

## Discussion

Overall, the sugar content of beverages available for purchase in Australia decreased by 1·3/100 ml (or 17 %) between 2015 and 2019. However, there was substantial heterogeneity across beverage categories, with large decreases observed in soft drinks and milk-based protein drinks, compensated by minimal change or even increases in other categories, such as fruit and vegetable juices, energy drinks, and ready-to-drink coffees. SSB have accounted for about 56 % of all beverages available for purchase since 2015. The reduction in sugar content among SSB was about 10 % (0·8 g/100 ml) and mainly driven by lowering the sugar content of soft drinks, milks and milk-based protein drinks. Taken together, these findings suggest the overall reduction in sugar content for all beverages was due to a combination of new low- or no-sugar products on the market and reformulation of some categories of SSB. Between 2015 and 2019, there was a 3 % increase in the proportion of beverages containing at least one NNS, more notably for waters, soft drinks and electrolyte/sports drinks. The decrease in sugar content for all beverages and SSB separately was, in general, larger for non-signatories than for signatories of the voluntary industry pledge from 2018, thus suggesting the pledge had little or no impact on lowering sugar content of beverages available for purchase in Australia.

The larger reduction in sugar observed for beverages overall (17 %) *v*. SSB alone (10 %) suggests at least some of the overall reduction is due to the appearance of new products with low or no sugar, rather than efforts to lower the sugar content of existing products. This is important because consumers may be reluctant to replace their usual beverage choices with low- or no-sugar substitutes, thus limiting the potential impact on population sugar intake^(21)^. In addition, SSB account for over half of all beverages available for purchase in Australia every year since 2015. Mean sugar content of SSB has declined only by 10 % between 2015 and 2019, suggesting only modest reformulation has occurred. However, the almost 25 % lowering in sugar content for SSB in the soft drinks category demonstrates reformulation is feasible and should be applied to other beverage categories. Our findings suggest that reformulation was achieved, at least in part, by adding NNS, as shown by the rise in the percentage of beverages containing NNS. Although NNS have minimal or no calories and are used in minute quantities, their long-term health effects remain controversial^(22)^, with emerging evidence suggesting they may have detrimental consequences at least for some individuals^(23)^. Therefore, while it is undoubtedly in the interest of public health to reduce intake of added sugars, their replacement by NNS should be cautious and complemented by policies that promote replacement of SSB by healthier alternatives, such as plain water or tea. A detailed analysis of the changes in NNS across the entire food and drink supply and its implications in Australia is available elsewhere^(24)^.

We found no evidence that the ABC’s voluntary sugar reduction pledge had led to a significant change in the sugar content of beverages available for purchase in Australia. Trends in sugar content for all drinks and SSB were broadly more favourable for manufacturers that did not sign the pledge than pledge signatories. This may be due to self-selection as the pledge was voluntary and, thus, companies with fewer SSB on their portfolio may have been more likely to sign the pledge than others. Furthermore, the decision by ABC to make the pledge apply retroactively from 2015 appeared to be an attempt to accrue credit from pre-existing reductions in sugar content in some cases, with our results showing that between 2015 and 2018, a substantial reduction in the mean sugar content of products available from pledge signatories had already occurred. It is, thus, germane to ask whether industry should have established a more ambitious lower target (i.e. a larger percentage reduction), or have used 2018 as a baseline rather than 2015. Our study casts doubt on signatories’ commitment to reduce the sugar content of their beverages and invest in reformulation of their SSB. In addition, the effectiveness of the pledge is limited as signatories only account for about 10 % of the beverages available for purchase in Australia, even if they may represent a larger proportion of sales. Indeed, Australia’s two largest non-alcoholic beverage bottlers, Coca-Cola Europacific Partners Australia and Asahi Holdings Australia Pty limited, are pledge signatories and represent 56·3 % of the market share for soft drinks and 85·7 % for bottled water^(25)^. Nonetheless, our findings suggest that voluntary sugar reduction initiatives will not drive consistent improvement across the entire beverage supply and are likely to have suboptimal public health impacts due to inherent conflicts between the public health objectives of such initiatives and the beverage industry’s profit imperative^(26)^. Food companies are often less committed to engage with sugar reduction programmes in the absence of a level playing field created by mandatory legislation, complete with sanctions or some form of financial incentive or penalty for lack of progress towards sugar reduction targets^(27)^. Commercial conflicts of interest are also likely to impair the achievement of the recently adopted government-endorsed voluntary targets for sugar reduction included in Australia’s Healthy Food Partnership initiative^(16)^.

By 2021, the Obesity Evidence Hub estimated that about fifty countries worldwide had implemented some form of tax on SSB despite wide variation in type and design of tax across countries (e.g. tiered *v*. uniform tax, eligible drinks, sugar limits, etc.)^(28)^. The benefits such taxes are fourfold: they introduce a price signal to consumers that a product is unhealthy; they create disincentives to purchase higher priced products; they provide profit incentives for manufacturers to systematically reformulate to reduce sugar content; and they generate revenue that governments can reinvest in public health and healthcare interventions^(29)^. Examples from across the world illustrate how those benefits can be achieved. In Mexico, a fixed tax of 1 peso (£0·03; €0·04; $0·04) per litre (approximately 10 % increase in price) was applied to all non-alcoholic drinks with added sugar starting in 2014. Two years after implementation of the tax, household purchases of taxed beverages decreased by an average of 7·6 %, with larger reductions in purchases documented in urban areas, in households with children and adolescents, in low socio-economic households, and among high SSB purchasing households^(30,31)^. In Portugal, the government introduced a consumption tax levied on SSB in 2017, which was divided into two tiers: drinks with sugar contents below 80 g/l of final product (charged at €8·22/100 l) are the lower tier and those above 80 g/l of final product (charged at €16·46/100 l) are the upper tier. During the first year of implementation, this tax collected about 80 million Euros, and all revenue was invested towards the Portuguese National Health Service funding. Although sales of SSB decreased by only about 7 %, reformulation processes led to an 11 % reduction of total energy intake through consumption of SSB at population level^(32)^. In response to these results, the Portuguese government redesigned the taxation to further encourage reformulation, but its evaluation is yet to be published^(33)^. In the UK, the Soft Drinks Industry Levy (SDIL) was implemented in 2018^(34)^. This two-tiered levy subjects high-sugar products to a higher tax than lower sugar products and led to a 10 % decrease in sugar consumption from soft drinks per household per week, despite no change in the volume of drinks purchased overall, thus demonstrating the potential to reduce the harmful effects of sugar intake without damaging industry profits^(35)^. The findings from individual countries have been recently confirmed by a systematic review and meta-analysis, which showed that the overall tax pass-through rate was 82 % and the demand for SSB was highly sensitive to tax-induced price increases, with a mean reduction in SSB sales of 15 %^(36)^. It did not find evidence of substitution to untaxed beverages or material changes in SSB consumption. Importantly, it demonstrated that tiered taxes resulted in reformulation and reduced sugar content of taxed beverages, whilst local-level taxes incentivised cross-border shopping, which are useful lessons for countries considering the pros and cons of different taxation models.

The mounting evidence supporting effective taxes on SSB elsewhere warrants consideration of the policy’s merits in Australia. Modelling by the Australian Medical Association in 2021 suggested a tax on selected SSB would reduce sugar consumption from soft drinks by 12–18 % and raise annual government revenue of $749 to $814 million^(37)^. This builds on prior research suggesting a 12·6 % decline in daily consumption of SSB could result in a decline in obesity of 2·7 % in men and 1·2 % in women over the lifetime^(38)^. It has also been estimated that a 20 % tax could save 1606 lives in 25 years, and over AUD$400 million in annual healthcare costs, almost 50 % of which would accrue in the most disadvantaged groups^(39)^. Furthermore, national surveys showed that 77 % of Australians supported a tax on sugary drinks if the proceeds were used to fund obesity prevention^(40)^.

Despite its myriad benefits, a tax on SSB is not a silver bullet that will address the excessive sugar intake and obesity epidemic in the Australian population^(41)^. Complementary policy-related measures that have been proposed include introducing package size limits^(42)^, improving information on added sugars on food labels, introducing mandatory targets or compositional limits on sugar content in SSB^(43)^, and restricting sale of SSB in some settings, such as schools, hospitals and workplaces^(44)^. Although a comprehensive suite of measures will likely be required to bolster ongoing progress in reducing sugar consumption in Australia, a tax on SSB would be a key step forward considering the compelling effectiveness of SSB taxes worldwide, which underpins the recommendation as one of the WHO ‘best-buy’ policies.

### Limitations

This study has some limitations. It lacked sales data and the ABC’s pledge was a commitment to reducing by 20 % sales-weighted sugar content, meaning our findings cannot directly assess achievement of the pledge overall. This means that even if there was a reduction in sugar content for some SSB, the intake of sugar accounted for by those drinks may have increased if consumption increased. Therefore, caution is needed when making inferences from our findings to impact on population sugar intake. Statistical tests were not performed as the FoodSwitch database represents more than 90 % of the beverage market in Australia, and hence statistical tests to account for sampling error would not likely have material impact on the interpretation of the study findings. As FoodSwitch data are captured at the same time each year, it is possible that seasonal products were missed. However, due to the transient nature of seasonal products in-store, it is unlikely that the inclusion/exclusion of these products would have changed the overall findings in the present study.

## Conclusions

Overall, there has been a 17 % reduction in sugar content of beverages available for purchase in Australia between 2015 and 2019, which resulted from a combination of new low- or no-sugar products and reformulation of pre-existing beverages. However, there is much progress to be made to curb the excessive consumption of sugar among the Australian population. The limited progress made by voluntary initiatives provides impetus for further consideration of mandatory legislative measures including a tax on SSB.

## Supplementary material

For supplementary material accompanying this paper visit https://doi.org/10.1017/S1368980022002300. click here to view supplementary material

## References

[1] Australian Institute of Health and Welfare (2018) Australia’s Health 2018. Australia’s Health Series no 16 AUS 221. Canberra: AIHW.

[2] Malik VS & Hu FB (2022) The role of sugar-sweetened beverages in the global epidemics of obesity and chronic diseases. Nat Rev Endocrinol 18, 205–218. 35064240 10.1038/s41574-021-00627-6 PMC8778490

[3] Australian Bureau of Statistics (2022) Australian Health Survey: Consumption of Added Sugars. https://www.abs.gov.au/statistics/health/health-conditions-and-risks/dietary-behaviour/2020-21 20 (accessed August 2022).

[4] Vartanian LR , Schwartz MB & Brownell KD (2007) Effects of soft drink consumption on nutrition and health: a systematic review and meta-analysis. Am J Public Health 97, 667–675. 17329656 10.2105/AJPH.2005.083782 PMC1829363

[5] de Ruyter JC , Olthof MR , Seidell JC et al. (2012) A trial of sugar-free or sugar-sweetened beverages and body weight in children. N Engl J Med 367, 1397–1406. 22998340 10.1056/NEJMoa1203034

[6] Malik VS , Pan A , Willett WC et al. (2013) Sugar-sweetened beverages and weight gain in children and adults: a systematic review and meta-analysis. Am J Clin Nutr 98, 1084–1102. 23966427 10.3945/ajcn.113.058362 PMC3778861

[7] Malik VS , Popkin BM , Bray GA et al. (2010) Sugar-sweetened beverages and risk of metabolic syndrome and type 2 diabetes. Diabetes Care 33, 2477–2483. 20693348 10.2337/dc10-1079 PMC2963518

[8] Xi B , Huang Y , Reilly KH et al. (2015) Sugar-sweetened beverages and risk of hypertension and CVD: a dose-response meta-analysis. Br J Nutr 113, 709–717. 25735740 10.1017/S0007114514004383

[9] Moynihan PJ & Kelly SA (2014) Effect on caries of restricting sugars intake: systematic review to inform WHO guidelines. J Dent Res 93, 8–18. 24323509 10.1177/0022034513508954 PMC3872848

[10] Te Morenga L , Mallard S & Mann J (2012) Dietary sugars and body weight: systematic review and meta-analyses of randomised controlled trials and cohort studies. BMJ 346, e7492. 23321486 10.1136/bmj.e7492

[11] National Health and Medical Research Council (2013) Australian Dietary Guidelines. Canberra: NHMRC.

[12] World Health Organization (2015) Guideline: Sugars Intake for Adults and Children. Geneva: WHO. 25905159

[13] Australian Bureau of Statistics (2016) Australian Health Survey: Consumption of Added Sugars 2011–2012 Report No. 4363.0.55.011. Canberra: Australian Bureau of Statistics.

[14] World Health Organization (2017) ‘Best Buys’ and Other Recommended Interventions for the Prevention and Control of Noncommunicable Diseases, Updated (2017) Appendix III of the Global Action Plan for the Prevention and Control of Non-Communicable Diseases 2013–2020. Geneva: WHO.

[15] Obesity Policy Coalition and GLOBE Obesity Centre (2017) Tipping the Scales: Australian Obesity Prevention Consensus. Melbourne: Obesity Policy Coalition and GLOBE Obesity Centre.

[16] Department of Health (2021) Healthy Food Partnership. Reformulation Targets. https://www1.health.gov.au/internet/main/publishing.nsf/Content/reformulation-targets (accessed October 2021).

[17] Australian Beverages Council (2018) Sugar Reduction Pledge. https://www.australianbeverages.org/initiatives-advocacy-information/sugar-reduction-pledge/ (accessed July 2019).

[18] Dunford E , Trevena H , Goodsell C et al. (2014) FoodSwitch: a mobile phone app to enable consumers to make healthier food choices and crowdsourcing of national food composition data. JMIR mHealth uHealth 2, e37. 25147135 10.2196/mhealth.3230 PMC4147708

[19] Dunford E , Webster J , Metzler AB et al. (2012) International collaborative project to compare and monitor the nutritional composition of processed foods. Eur J Prev Cardiol 19, 1326–1332. 21971487 10.1177/1741826711425777

[20] Australia New Zealand Food Standards Code (2022) Standard 1.2.7 Nutrition, Health and Related Claims (Cth).https://www.foodstandards.gov.au/code/Documents/1.2.7%20Nutrition%20and%20health%20claims%20v157.pdf (accessed May 2022)

[21] Fichera E , Mora T , Lopez-Valcarcel BG et al. (2021) How do consumers respond to “sin taxes”? New evidence from a tax on sugary drinks. Soc Sci Med 274, 113799. 33684702 10.1016/j.socscimed.2021.113799

[22] Mooradian AD , Smith M & Tokuda M (2017) The role of artificial and natural sweeteners in reducing the consumption of table sugar: a narrative review. Clin Nutr ESPEN 18, 1–8. 29132732 10.1016/j.clnesp.2017.01.004

[23] Suez J , Cohen Y , Valdés-Mas R et al. (2022) Personalized microbiome-driven effects of non-nutritive sweeteners on human glucose tolerance. Cell 185, 3307.e19–3328.e19. 35987213 10.1016/j.cell.2022.07.016

[24] Dunford EK , Coyle DH , Louie JCY et al. (2022) Changes in the presence of nonnutritive sweeteners, sugar alcohols, and free sugars in Australian foods. J Acad Nutr Diet 122, 991.e7–999.e7. 34864247 10.1016/j.jand.2021.11.018

[25] KPMG Australia (2020) Sugar Reduction Pledge by the Australian Non-Alcoholic Drinks Industry 2020 Aggregation Report. https://www.australianbeverages.org/wp-content/uploads/2020/10/Second-progress-report_Sugar-reduction-pledge_F_21102020.pdf (accessed September 2021).

[26] Knai C , Petticrew M , Douglas N et al. (2018) The public health responsibility deal: using a systems-level analysis to understand the lack of impact on alcohol, food, physical activity, and workplace health sub-systems. Int J Environ Res Public Health 15, 2895. 30562999 10.3390/ijerph15122895 PMC6313377

[27] Bandy LK , Scarborough P , Harrington RA et al. (2021) The sugar content of foods in the UK by category and company: a repeated cross-sectional study, 2015–2018. PLoS Med 18, e1003647. 34003863 10.1371/journal.pmed.1003647 PMC8171925

[28] Obesity Prevention Hub Countries that Have Taxes on Sugar-Sweetened Beverages (SSBs). https://www.obesityevidencehub.org.au/collections/prevention/countries-that-have-implemented-taxes-on-sugar-sweetened-beverages-ssbs (accessed September 2021).

[29] Wright A , Smith KE & Hellowell M (2017) Policy lessons from health taxes: a systematic review of empirical studies. BMC Public Health 17, 583. 28629470 10.1186/s12889-017-4497-z PMC5477308

[30] Colchero MA , Molina M & Guerrero-López CM (2017) After Mexico implemented a tax, purchases of sugar-sweetened beverages decreased and water increased: difference by place of residence, household composition, and income level. J Nutr 147, 1552–1557. 28615377 10.3945/jn.117.251892 PMC5525113

[31] Ng SW , Rivera JA , Popkin BM et al. (2018) Did high sugar-sweetened beverage purchasers respond differently to the excise tax on sugar-sweetened beverages in Mexico? Public Health Nutr 22(4), 750–756. 30560754 10.1017/S136898001800321X PMC6581622

[32] Goiana-da-Silva F , Nunes AM , Miraldo M et al. (2018) Using pricing policies to promote public health: the sugar sweetened beverages taxation experience in Portugal. Acta Med Port 31, 191–195. 29855411 10.20344/amp.10222

[33] Goiana-da-Silva F , Cruz-e-Silva D , Gregório MJ et al. (2018) The future of the sweetened beverages tax in Portugal. Lancet Public Health 3, e562. 30522681 10.1016/S2468-2667(18)30240-8

[34] Bandy LK , Scarborough P , Harrington RA et al. (2020) Reductions in sugar sales from soft drinks in the UK from 2015 to 2018. BMC Med 18, 20. 31931800 10.1186/s12916-019-1477-4 PMC6956503

[35] Pell D , Mytton O , Penney TL et al. (2021) Changes in soft drinks purchased by British households associated with the UK soft drinks industry levy: controlled interrupted time series analysis. BMJ 372, n254. 33692200 10.1136/bmj.n254 PMC7944367

[36] Andreyeva T , Marple K , Marinello S et al. (2022) Outcomes following taxation of sugar-sweetened beverages: a systematic review and meta-analysis. JAMA Netw Open 5, e2215276. 35648398 10.1001/jamanetworkopen.2022.15276 PMC9161017

[37] Australian Medical Association (2021) A Tax on Sugar-Sweetened Beverages: Modelled Impacts on Sugar Consumption and Government Revenue. Melbourne: Australian Medical Association.

[38] Veerman JL , Sacks G , Antonopoulos N et al. (2016) The impact of a tax on sugar-sweetened beverages on health and health care costs: a modelling study. PLoS ONE 11, e0151460. 27073855 10.1371/journal.pone.0151460 PMC4830445

[39] Lal A , Mantilla-Herrera AM , Veerman L et al. (2017) Modelled health benefits of a sugar-sweetened beverage tax across different socioeconomic groups in Australia: a cost-effectiveness and equity analysis. PLoS Med 14, e1002326. 28654688 10.1371/journal.pmed.1002326 PMC5486958

[40] Falbe J , Thompson HR , Becker CM et al. (2016) Impact of the Berkeley excise tax on sugar-sweetened beverage consumption. Am J Public Health 106, 1865–1871. 27552267 10.2105/AJPH.2016.303362 PMC5024386

[41] von Philipsborn P , Stratil JM , Burns J et al. (2019) Environmental interventions to reduce the consumption of sugar-sweetened beverages and their effects on health. Cochrane Database Syst Rev 6, CD012292. 31194900 10.1002/14651858.CD012292.pub2 PMC6564085

[42] Crino M , Herrera AMM , Ananthapavan J et al. (2017) Modelled cost-effectiveness of a package size cap and a kilojoule reduction intervention to reduce energy intake from sugar-sweetened beverages in Australia. Nutrients 9, 983. 28878175 10.3390/nu9090983 PMC5622743

[43] Food Regulation (2021) Policy Paper: Exploring Options for Improving the Composition of the Food Supply. https://foodregulation.gov.au/internet/fr/publishing.nsf/Content/policy-paper-food-supply-composition (accessed August 2021).

[44] Epel ES , Hartman A , Jacobs LM et al. (2020) Association of a workplace sales ban on sugar-sweetened beverages with employee consumption of sugar-sweetened beverages and health. JAMA Intern Med 180, 9–16. 31657840 10.1001/jamainternmed.2019.4434 PMC6820289

