# Supplementary material for: Trends in sugar content of non-alcoholic beverages in Australia between 2015 and 2019 during the operation of a voluntary industry pledge to reduce sugar content
Source: Public Health Nutr. 2022 Oct 24;26(1):287–96. doi: 10.1017/S1368980022002300 (PMC11077450; doi:10.1017/S1368980022002300)
Supplement: Supplementary file 1 [file S1368980022002300sup001.docx]

**Supplementary data**

**Table S1: Included and excluded beverage categories**

| Included | Definition | Excluded |
| --- | --- | --- |
| Breakfast beverages | Beverages containing variable combinations of dairy or non-dairy milk, coffee, cereals and fruit, including smoothies | Instant coffee powder and pods, coffee whitener |
| Coffee – ready-to-drink coffee products |  | Drinking yoghurt |
| Cordials/syrups | Sweet, fruit-flavoured drinks | Evaporated, condensed and powdered milk |
| Electrolyte drinks | Beverages containing water, electrolytes (usually sodium and potassium), and sugar or sweeteners | Hot chocolate |
| Energy drinks | Beverages containing a high percentage of sugar or sweetener, caffeine, or another stimulant | Other flavourings for milk |
| Fruit and vegetable juices |  | Probiotic drinks |
| Milk (dairy or non-dairy, plain or flavoured) |  | Raw cocoa powder |
| Milk-based protein drinks | Beverages containing milk-based protein powder and variable combinations of vegetable oils, carbohydrates, vitamins, minerals and trace-elements | Tea |
| Soft drinks | Beverages containing water (often carbonated), sugar or sweetener, and a natural and/or artificial flavouring (e.g., iced teas, Coca Cola, Pepsi Co, etc.) |  |
| Water (plain or flavoured) |  |  |
|  |  |  |
|  |  |  |
|  |  |  |
|  |  |  |
|  |  |  |

**Table S2: Search terms for added sugars**

| Added sugars |
| --- |
| agave |
| corn sweetener |
| dextrose |
| fermented cane spirit |
| fructose |
| glucose |
| glucose-liquid |
| honey |
| hopped malt extract |
| lactose |
| liquid malt extract |
| maltose |
| malt barley extract |
| maltodextrin |
| molasses |
| sucrose |
| sugar |
| syrup |
| treacle |
| white icing |

**Table S3: Search terms for non-nutritive sweeteners and sugar alcohols**

| Non-nutritive sweeteners | Search terms |
| --- | --- |
| Acesulfame K | Acesulfame K, Equal, Twinsweet, Sunett, Sweet one, E950, E962 |
| Alitame | Alitame, E956 |
| Aspartame | Aspartame, Equal, Instasweet, Natrataste, Nutrasweet, Twinsweet, E951, E962 |
| Cyclamate | Cyclamate, E952 |
| Monkfruit extract | Monkfruit, Luohan guo |
| Neotame | Neotame, Newtame, E961 |
| Saccharin | Saccharin, Nectasweet, Sugartwin, Sweet'n low, E954 |
| Steviol glycosides | Steviol glycosides, Enliten, Candyleaf, Rebaudioside A, Reb-a, Rebiana, Sugarleaf, Sweetleaf, Truvia, Stevia, Purevia, E960 |
| Sucralose | Sucralose, Altern, Kaltame, Splenda, Trichlorogalactosucrose, E955 |
| Thaumatin | Thaumatin, E957 |
| Sugar alcohols | **Search terms** |
| Erythritol | Erythritol, E968 |
| Isomalt | Isomalt, E953 |
| Lactitol | Lactitol, E966 |
| Maltitol | Maltitol, E965 |
| Sorbitol | Sorbitol, E420 |
| Mannitol | Mannitol, E961 |
| Polyglycitol | Polyglycitol, E964 |
| Xylitol | Xylitol, E967 |

**Table S4: Availability of beverages, stratified by category, in Australia between 2015 and 2019 (N and (%))**

| **Beverage categories** | **2015** | **2016** | **2017** | **2018** | **2019** |
| --- | --- | --- | --- | --- | --- |
| **Breakfast beverages** | **42 (2.8)** | **26 (1.7)** | **20 (1.3)** | **33 (1.9)** | **31 (1.9)** |
| **Ready-to-drink coffees** | **61 (4.1)** | **56 (3.7)** | **49 (3.3)** | **62 (3.70** | **54 (3.3)** |
| **Cordials/syrups** | **99 (6.6)** | **90 (5.9)** | **68 (4.5)** | **67 (4.1)** | **56 (3.5)** |
| Sugar-free | 19 (1.3) | 16 (1.1) | 10 (0.7) | 8 (0.5) | 11 (0.7) |
| Sugar-sweetened | 80 (5.3) | 74 (4.9) | 58 (3.9) | 59 (3.6) | 45 (2.8) |
| **Electrolyte drinks** | **26 (1.7)** | **29 (1.9)** | **36 (2.4)** | **47 (2.8)** | **43 (2.7)** |
| Sugar-free | 1 (0.1) | 3 (0.2) | 5 (0.3) | 8 (0.5) | 8 (0.5) |
| Sugar-sweetened | 25 (1.7) | 26 (1.7) | 31 (2.1) | 39 (2.4) | 35 (2.2) |
| **Energy drinks** | **40 (2.7)** | **46 (3.1)** | **47 (3.1)** | **46 (2.8)** | **48 (2.9)** |
| Sugar-free | 13 (0.9) | 15 (1.0) | 15 (1.0) | 17 (1.0) | 12 (0.7) |
| Sugar-sweetened | 27 (1.8) | 31 (2.1) | 32 (2.1) | 29 (1.7) | 36 (2.2) |
| **Fruit and vegetable juices** | **523 (34.9)** | **484 (32.2)** | **470 (31.3)** | **418 (25.3)** | **401 (24.8)** |
| Fruit juice | 498 (33.2) | 453 (30.1) | 435 (28.9) | 388 (23.4) | 379 (23.5) |
| Vegetable juice | 25 (1.7) | 31 (2.1) | 35 (2.3) | 30 (1.8) | 22 (1.4) |
| **Milk** | **243 (16.2)** | **266 (17.7)** | **280 (18.6)** | **317 (19.1)** | **331 (20.5)** |
| Coconut milk | 4 (0.3) | 5 (0.3) | 12 (0.8) | 15 (0.9) | 11 (0.7) |
| Dairy milk | 165 (11.0) | 177 (11.8) | 185 (12.3) | 197 (11.9) | 214 (13.3) |
| Lactose-free milk | 7 (0.5) | 10 (0.7) | 10 (0.7) | 11 (0.7) | 15 (0.9) |
| Soy milk | 28 (1.9) | 30 (2.0) | 30 (2.0) | 29 (1.8) | 30 (1.9) |
| Other milk | 39 (2.6) | 44 (2.9) | 43 (2.9) | 65 (3.9) | 61 (3.8) |
| **Milk-based protein drinks** | **15 (1.0)** | **18 (1.2)** | **16 (1.1)** | **37 (2.2)** | **20 (1.2)** |
| **Soft drinks** | **277 (18.5)** | **284 (18.9)** | **299 (19.9)** | **366 (22.1)** | **388 (24.0)** |
| Sugar-free | 53 (3.5) | 55 (3.7) | 44 (2.9) | 63 (3.8) | 76 (4.7) |
| Sugar-sweetened | 224 (14.9) | 229 (15.2) | 255 (16.9) | 303 (18.3) | 312 (19.3) |
| **Waters** | **145 (9.7)** | **169 (11.2)** | **184 (12.3)** | **223 (13.5)** | **220 (13.6)** |
| Aloe vera drinks | 8 (0.5) | 9 (0.6) | 10 (0.7) | 11 (0.7) | 13 (0.8) |
| Coconut water | 28 (1.9) | 40 (2.7) | 55 (3.7) | 49 (2.9) | 42 (2.6) |
| Flavoured waters | 33 (2.2) | 40 (2.7) | 45 (3.0) | 65 (3.9) | 52 (3.2) |
| Sparkling water, plain | 33 (2.2) | 38 (2.5) | 37 (2.5) | 44 (2.7) | 67 (4.2) |
| Still water, plain | 43 (2.9) | 42 (2.8) | 37 (2.5) | 54 (3.3) | 46 (2.9) |
| **Total** | **1499** | **1503** | **1502** | **1655** | **1615** |

**Table S5: Percentage of beverages with at least one non-nutritive sweetener, stratified by category, available for purchase in Australia between 2015 and 2019**

| **Beverage categories** | **2015** | **2016** | **2017** | **2018** | **2019** |
| --- | --- | --- | --- | --- | --- |
| **Breakfast beverages** | **4.8** | **11.5** | **0.0** | **0.0** | **3.2** |
| **Ready-to-drink coffees** | **8.2** | **5.4** | **0.0** | **3.2** | **1.9** |
| **Cordials/syrups** | **59.6** | **51.1** | **44.1** | **44.8** | **53.6** |
| Sugar-free | 100.0 | 100.0 | 100.0 | 100.0 | 100.0 |
| Sugar-sweetened | 50.0 | 40.5 | 34.5 | 37.3 | 42.2 |
| **Electrolyte drinks** | **11.5** | **20.7** | **22.2** | **17.0** | **32.6** |
| Sugar-free | 0.0 | 100.0 | 100.0 | 75.0 | 100.0 |
| Sugar-sweetened | 12.0 | 11.5 | 9.7 | 5.1 | 17.1 |
| **Energy drinks** | **42.5** | **43.5** | **44.7** | **52.2** | **41.7** |
| Sugar-free | 100.0 | 100.0 | 100.0 | 100.0 | 100.0 |
| Sugar-sweetened | 14.8 | 16.1 | 18.8 | 24.1 | 22.2 |
| **Fruit and vegetable juices** | **3.3** | **3.1** | **2.1** | **4.1** | **5.0** |
| Fruit juice | 3.4 | 3.3 | 2.3 | 4.4 | 5.3 |
| Vegetable juice | 0.0 | 0.0 | 0.0 | 0.0 | 0.0 |
| **Milk** | **2.1** | **3.4** | **4.3** | **3.8** | **3.6** |
| Coconut milk | 0.0 | 0.0 | 0.0 | 0.0 | 0.0 |
| Dairy milk | 3.0 | 4.5 | 6.5 | 5.1 | 3.7 |
| Lactose-free milk | 0.0 | 0.0 | 0.0 | 0.0 | 0.0 |
| Soy milk | 0.0 | 2.3 | 0.0 | 3.1 | 6.6 |
| Other milk | 0.0 | 0.0 | 0.0 | 0.0 | 0.0 |
| **Milk-based protein drinks** | **93.3** | **94.4** | **93.8** | **91.9** | **95.0** |
| **Soft drinks** | **31.0** | **29.9** | **33.8** | **41.8** | **48.5** |
| Sugar-free | 96.2 | 98.2 | 97.7 | 100.0 | 98.7 |
| Sugar-sweetened | 15.6 | 13.5 | 22.7 | 29.7 | 36.2 |
| **Waters** | **3.4** | **4.1** | **3.8** | **10.3** | **13.2** |
| Aloe vera drinks | 12.5 | 0.0 | 10.0 | 27.3 | 23.1 |
| Coconut water | 0.0 | 0.0 | 0.0 | 0.0 | 2.4 |
| Flavoured waters | 12.1 | 17.5 | 13.3 | 30.8 | 40.4 |
| Sparkling water, plain | 0.0 | 0.0 | 0.0 | 0.0 | 4.5 |
| Still water, plain | 0.0 | 0.0 | 0.0 | 0.0 | 2.2 |
| **Total** | **41.2** | **41.1** | **40.8** | **42.4** | **44.3** |

**Figure S1: Percentage change in sugar content (per 100mL) for pledge signatories versus non-signatories between 2015 and 2018 for all beverages (A) and sugar-sweetened beverages (B)**

**A**

**B**
